# Sexual dimorphism in the nutritional requirement for adult lifespan in *Drosophila melanogaster*

**DOI:** 10.1111/acel.13120

**Published:** 2020-02-18

**Authors:** Qi Wu, Guixiang Yu, Xingyi Cheng, Yue Gao, Xiaolan Fan, Deying Yang, Meng Xie, Tao Wang, Matthew D. W. Piper, Mingyao Yang

**Affiliations:** ^1^ Institute of Animal Genetics and Breeding Sichuan Agricultural University Chengdu China; ^2^ School of Biological Sciences Monash University Clayton Vic. Australia

**Keywords:** *Drosophila*, lifespan, nutritional requirement, sexual dimorphism

## Abstract

The nutritional requirements of *Drosophila* have mostly been studied for development and reproduction, but the minimal requirements for adult male and female flies for lifespan have not been established. Following development on a complete diet, we find substantial sex difference in the basic nutritional requirement of adult flies for full length of life. Relative to females, males require less of each nutrient, and for some nutrients that are essential for development, adult males have no requirement at all for lifespan. The most extreme (and surprising) sex differences were that chronic cholesterol and vitamin deficiencies had no effect on the lifespan of adult males, but they greatly decreased lifespan in females. Female oogenesis rather than chromosomal karyotype and mating status is the key cause of this gender difference in life‐sustaining nutritional requirements. These data are important to the way we understand the mechanisms by which diet modifies lifespan.

## Graphical abstract

We found that relative to females, adult males require less of each nutrient for lifespan. The most surprising sex differences was that chronic deprivation of the developmentally essential nutrients cholesterol and vitamins had no effect on the lifespan of adult males, but they greatly decreased lifespan in females. Female oogenesis rather than chromosomal karyotype and mating status is the key cause of this gender difference in life‐sustaining nutritional requirements.

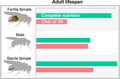

## 1 INTRODUCTION

Organisms have evolved under conditions in which their available nutrition does not perfectly match their requirements (Simpson & Raubenheimer, 2012). This mismatch means that some nutrients are undersupplied relative to demand, and so in order to thrive, organisms must selectively invest these limiting resources among competing physiological demands (Maklakov & Immler, 2016; Simpson & Raubenheimer, 2012). This important concept of resource allocation lies at the heart of theoretical attempts to explain how moderate dietary restriction (DR) or altered diet balance (DB) can operate to modify lifespan (Kirkwood, 1977; Williams, 1966). In these models, some diets promote greater resource allocation to “somatic maintenance”—a subunit of physiology that preserves the adult soma for longer life—at the expense of investment in reproduction (Maklakov & Immler, 2016). Many theories have been proposed to define the processes underlying somatic maintenance, particularly in the field of research on aging (Kirkwood, 2002; Maklakov & Immler, 2016; Shanley & Kirkwood, 2000), but the limits of its nutritional requirements have not been defined.

*Drosophila melanogaster* has been used as a model for nutritional physiology studies for many years (Piper, 2017; Rauser, Mueller, & Rose, 2004; Tatar, Post, & Yu, 2014), and their requirements for development and female reproduction are well studied (Begg & Robertson, 1950; Consuegra et al., 2019; Piper, 2017; Sang & King, 1961). We also know that the lifespan of male and female adults respond differently to dietary interventions and that the sexes show different preference in macronutrient balance (Bowman & Tatar, 2016; Camus, Huang, Reuter, & Fowler, 2018; Chandegra, Tang, Chi, & Alic, 2017; Lee, Kim, & Min, 2013; Magwere, Chapman, & Partridge, 2004; Regan et al., 2016; Wu et al., 2020), but no one has systematically determined the minimal requirements of each nutrient class for adult lifespan and whether these requirements differ between the sexes. Defining these limits is important since it is fundamental to understanding how adult‐specific diet interventions, such as DR and DB, may be operating to modify lifespan. Here, we uncover substantial sex differences in the basic nutritional requirement of adult lifespan in *Drosophila*.

## 2 RESULTS

### 2.1 Nutritional requirements for sustaining life are much lower in males than in females

**Figure 1:**
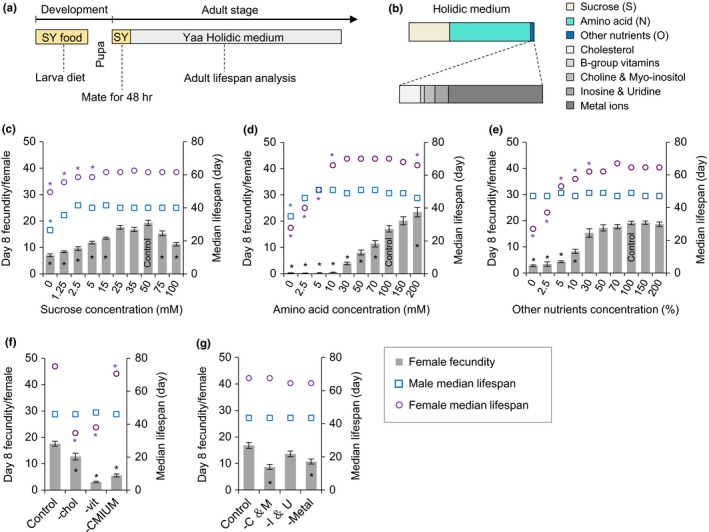
Sex difference in basic nutritional requirement for lifespan in *Drosophila*. (a) Experimental timeline for rearing and exposure of adult flies to experimental diets. (b) Outline of the nutritional composition of the chemically defined (holidic) medium. (c‐e) Effect of manipulating sugar (c), total amino acids (d), and other nutrients levels (e) on female day 8 fecundity and median lifespan in both sexes. The effect of diets and sex on lifespan, and their interactions, was significant in all nutrient manipulations (*p* < .001, Cox regression). (f) Effects of omitting dietary cholesterol (chol), B‐group vitamins (vit), or other ingredients include choline, myo‐inositol, inosine, uridine, and metal ions (labeled CMIUM) on female day 8 fecundity and lifespan in both sexes. These showed that male and female lifespan responded differently to omitting cholesterol or vitamins but not to CMIUM omission. (g) Effect of omitting choline and myo‐inositol (C and M), inosine and uridine (I and U), or metal ions on female day 8 fecundity and lifespan in both sexes. All treatments had no effect on lifespan in both males and females. (*n* = 100 wild‐type *Dahomey* flies per treatment for lifespans and *n* = 10 biological replicates for egg laying in all trials, error bars represent egg‐laying mean ± *SEM*). Within each phenotype,* in the figure indicate *p* < .05 versus complete holidic diet control. Lifespan differences were assessed using the Cox regression model, and egg‐laying differences were assessed by one‐way ANOVA followed by Tukey's multiple comparison. See statistical analysis of lifespan data in Tables S1‐S5)

To investigate sex‐specific nutritional requirements for adult lifespan in *Drosophila*, we used the holidic medium described in Piper et al. (2014), in which the relative abundance of most nutrients, including the amino acid ratio, follows the pattern found in yeast (Yaa) (Figure 1a,b). We independently manipulated the concentration of each nutrient class and measured female fecundity as well as male and female lifespan. All flies were reared from egg to adulthood on a complete sugar/yeast (SY) medium, in the same way as is done for all DR and DB lifespan studies (Bass et al., 2007). This is done to standardize the condition of all adults to a high level before they receive dietary treatments. Once emerged, adult flies were allowed to mate for 48 hr on the same SY medium before the sexes were separated for lifelong monitoring of fecundity and survival on the various modified holidic media (Figure 1a,b).

Sucrose and amino acids make up about 97% of all nutrients in the holidic medium as a molar fraction and are the sole source of carbohydrates and protein equivalents, which on standard diets are known to be the major drivers of fitness traits (Lee et al., 2008; Mair, Piper, & Partridge, 2005; Skorupa, Dervisefendic, Zwiener, & Pletcher, 2008) (Figure 1b). Therefore, we first investigated the lifespan of male and female flies when only these two components of the medium were varied and all others remained fixed.

In response to changing the concentration of sugar only (Figure 1c and Figure S1a), female egg laying increased as sugar concentration increased from 0–25 mM and stayed at its maximum level up to 50 mM. As previously observed, any further increase in sugar concentration caused egg laying to decrease (Piper et al., 2014). At low concentrations of sugar, lifespan of both sexes was significantly compromised. Using both the log‐rank test and Cox regression to compare lifespan curves, we found that the minimal requirement for sugar to maintain maximal lifespan in males is 1.25 mM; this concentration supported maximal lifespan, and further additions were of no added benefit (Figure 1c and Figure S1a and Table S1). For mated females, a sugar concentration between 5 mM and 15 mM was required to support maximal lifespan (Figure 1c and Figure S1a and Table S1). The physiological effect of removing all carbohydrates from the food was relatively modest, since lifespan was only reduced to 80% of its maximum in females and 66% in males, and egg laying dropped to ~ 50% of its maximum (Figure 1c). These data indicate that the need for sucrose for providing energy and a carbon backbone for various macromolecules can be supplanted by other nutrients, most likely dietary amino acids.

In response to changes in the concentration of the amino acid mixture (expressed as N, which represents the sum of theoretically bioavailable nitrogen in mM if all amino acids were fully catabolized) (Figure 1d and Figure S1b), female flies showed a dramatic egg lay and lifespan response. From 0 to 10 mM total dietary N, females exhibited negligible egg laying, while lifespan approximately doubled (28 days median to 66 days median). Interestingly, egg laying only showed a noticeable increase once the N requirement for maximal lifespan was met (~30 mM total N). Thus, females appear to prioritize the use of low amounts of dietary amino acids for somatic maintenance at the expense of reproduction. This is different from their response to low amounts of sugar for which increasing doses promote both lifespan and egg laying at the same time. Additions of N from 30 mM to 200 mM drove ever increasing egg laying, while lifespan remained at its maximum from 30 mM to 100 mM N. As N rose from 100 mM to 200 mM, N female lifespan was reduced, even though N remained limiting for egg laying. Together, this pattern is consistent with a model in which the flies continuously invest amino acids to maintain the soma at maximal health, channeling only surplus amino acids into reproduction. However, very high levels of amino acids shorten lifespan, not because of an insufficiency for somatic maintenance, but because of some damaging effect of long‐term exposure to high amounts (Fanson, Fanson, & Taylor, 2012; Piper et al., 2017). Males exhibited an approximately fivefold to 10‐fold lower minimal requirement for dietary amino acids for maximal lifespan (2.5 mM N) than did females (10–30 mM N) indicating an extremely low protein requirement to support male life.

When sugar and amino acids were fixed at the levels that maximized lifespan and a dilution series of all other nutrients was performed, we saw that some component(s) were limiting for both egg laying and female lifespan up to 30% of the amount in the control diet (Figure 1e and Figure S1c). Surprisingly, even though this mixture contains nutrients that flies cannot synthesize de novo, males exhibited no requirement at all for any nutrient other than sugar and amino acids, for maintaining full adult lifespan. The finding that none of these components were required in the adult diet of males was evident whether they were omitted from the diet as a group (Figure 1e and Figure S1c) or individually (Figure 1f,g and Figure S1d,e and Figure S3a,b).

In order to investigate further the female requirement for the nonenergy yielding nutrients, we dropped out individual components and assessed reproduction and lifespan responses. Consistent with previous reports, omitting cholesterol or vitamins from adult diets significantly decreased both lifespan and egg laying of females ([Piper et al., 2014]; Figure 1f and Figure S1d), a finding that we replicated in two other laboratory strains of *Drosophila* (w1118 and Canton S; Figure S3a,b). Removing the remaining components (choline, myo‐inositol, uridine, inosine, and metals) as a mixture also reduced female fecundity and slightly decreased female lifespan (Figure 1f and Figure S1d). Further work revealed that removing choline and myo‐inositol (C and M) or metal ions, but not inosine and uridine (I and U) reduced egg laying (Figure 1g), which is consistent with the fly's predicted ability to synthesize these components de novo (Piper, 2017). Interestingly, removing any of these three components in isolation did not reduce longevity in females indicating the flies can identify their absence from the diet and preferentially retain these for somatic maintenance at the expense of reproduction (Figure 1g and Figure S1e).

### 2.2 Sex chromosome complement and mating status do not explain the sexual dimorphism of adult nutritional requirement

**Figure 2:**
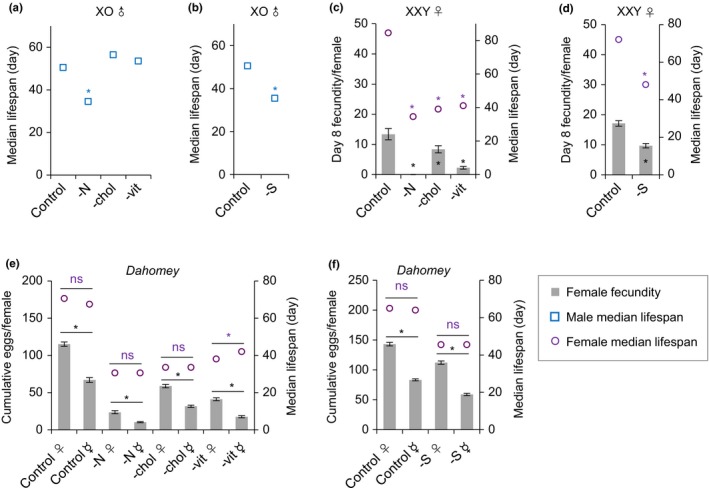
Sex chromosome and mating status have no effect on nutritional requirement for lifespan in *Drosophila*. (a and b) Effect of omitting amino acids (N), cholesterol (chol), B‐group vitamins (vit), or sucrose (S) on median lifespan of XO males. (c and d) Effect of omitting N, chol, vit, or S on day 8 fecundity and median lifespan of XXY females. (e and f) Effect of omitting N, chol, vit, or S on the lifetime cumulative egg number and median lifespan of mated wild‐type *Dahomey* females (♀) and virgin females (☿). Virgin females exhibit a similar baseline requirement for sugar, amino acids, and cholesterol relative to mated females, but show a slight survival advantage in vitamins deficient diet.(*n* = 100 flies per treatment for lifespans and *n* = 10 biological replicates for egg laying in all trials, error bars represent egg‐laying mean ± s.e.m). Within each phenotype,* indicate *p* < .05 versus complete holidic diet control. Lifespan differences were assessed using the Cox regression model. Egg‐laying differences in panel c were assessed by one‐way ANOVA followed by Tukey's multiple comparison; egg‐laying differences in panel d were assessed by unpaired *t* test. Egg‐laying differences in panel e‐f were assessed by two‐way ANOVA followed by Sidak's multiple comparisons test. See statistical analysis of lifespan data in Tables S6‐S11)

Recent studies indicate that sex chromosomes underlie at least some of the sex difference in lifespan in mice (Davis, Lobach, & Dubal, 2018). To assess whether the dosage of sex chromosomes modifies the basic nutritional requirements of the sexes in flies, we generated males lacking a Y chromosome (XO; sterile) as well as females carrying a Y chromosome (XXY; fertile). We found that although XO males are sterile, the pattern of change in lifespan in response to the absence of amino acids, sugar, cholesterol, or vitamins (Figure 2a,b and Figure S2a,b) was not different from that of fertile XY Dahomey males (Figure 1). These data indicate that the cost to lifespan of gamete production in fertile males is extremely low and seemingly unaffected by adult nutrition. Similarly, the changes in lifespan for XXY females (Figure 2c,d and Figure S2c,d) followed the same pattern as Dahomey XX females (Figure 1). Thus, differences in the complement of sex chromosomes did not change the nutritional requirements of flies for fecundity and lifespan. Instead, this sexual dimorphism appears to be caused by the different investment in reproduction between males and females.

One of the major differences between the sexes in our experiments is that the females experience on ongoing cost of reproduction through continuous egg laying, whereas the males did not since they were maintained apart from females except for the 48‐hr window immediately after eclosion. To assess whether a reduced cost of mating would modify the female flies' nutritional requirements for lifespan to resemble that of males, we compared mated females to virgin females. Virgin females have a reduced cost of mating both because they lay fewer eggs and they do not suffer from the ongoing consequences of mating induced harm (Salmon, Marx, & Harshman, 2001). Except for when vitamins were omitted, which resulted in virgins still having short lives but slightly longer than mated flies, the requirements of virgins for each of the essential nutrient dropouts tested were not different from that of mated females (Figure 2e,f and Figure S2e,f). Thus, female flies exhibit a similar baseline requirement for sugar, amino acids, cholesterol, and vitamins for lifespan whether or not they were mated.

### 2.3 Female oogenesis is the key cause of the gender difference in life‐sustaining nutritional requirements

**Figure 3:**
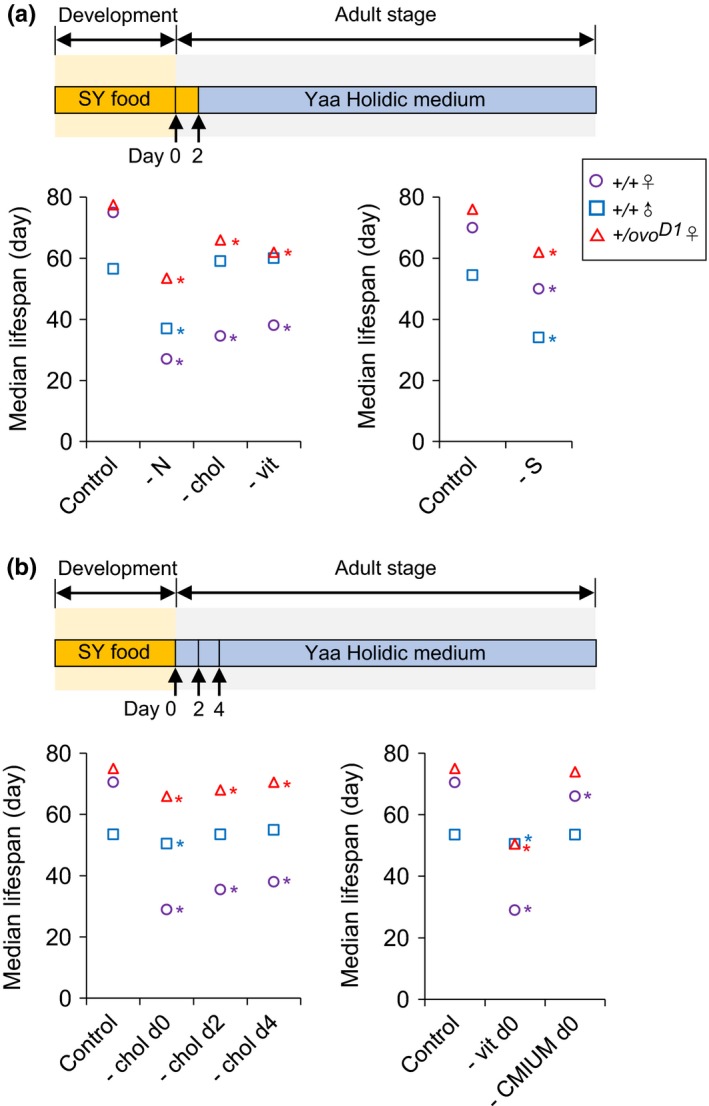
*ovo^D1^* mutation greatly rescued female lifespan in malnutrition diets. (a) Effect of omitting amino acids (N), cholesterol (chol), B‐group vitamins (vit), or sucrose (S) from the adult diet from day 2 posteclosion on median lifespan of wild‐type females and males and *ovo^D1^* mutant females. For fertile females and *ovo^D1^* sterile females, there was a significant diet × genotype interaction due to *ovo^D1^* sterile females having a reduced requirement for amino acids, cholesterol, and vitamins relative to fertile females. Males also had a reduced requirement for these nutrients when compared to fertile females. (b) Effect of omitting cholesterol in adult diet from days 0, 2, and 4 posteclosion on median lifespan of wild‐type females and males and *ovo^D1^* mutant females (left panel). And effect of omitting vitamins or choline, myo‐inositol, uridine, inosine, and metals (CMIUM) in adult diet immediately from posteclosion on median lifespan of wild‐type females and males and *ovo^D1^* mutant females (right panel). (* in the figure indicate *p* < .05 vs. complete holidic diet control in each genotype, Cox regression. *n* = 100 flies per treatment for lifespans, see statistical analysis of lifespan data in Tables S14‐S18)

Although virgins have a reduced cost of mating, virgins of our strain of *Drosophila*, Dahomey, still lay eggs at ~ 50% of the level of mated flies. To assess whether the cost to lifespan in virgins was due to the investment of dietary nutrients into oogenesis, we reared *ovo^D1^* mutant females in which oogenesis is blocked at stage 4, meaning these flies are spared from the substantial resource investment into eggs at the yolking stages (Granadino, San Juan, Santamaria, & Sanchez, 1992) as well as avoiding any physical costs of laying eggs. Under fully fed conditions, *ovo^D1^* mutant females were longer lived than fertile female controls as has been previously reported (Mair, Sgro, Johnson, Chapman, & Partridge, 2004). Furthermore, in the absence of either amino acids, sugar, cholesterol, or vitamins, *ovo^D1^* mutants were substantially longer lived than fertile females, but still not as long lived as when they were fully fed (Figure 3a, Figure S4a‐c). These same outcomes appear specific to infertility since we observed the same effects for *ovo^D1^* mutant females in another genetic background (Figure S5). Thus, adult females require these nutrients in the diet for survival whether or not they are producing eggs. In contrast, and just like males, *ovo^D1^* sterile females did not require dietary choline, myo‐inositol, inosine, uridine, or metal ions (Figure 3b and Figure S4f) demonstrating that adult supplementation of these nutrients is only needed for oogenesis.

Our rearing protocol for lifespan experiments allows all flies to develop on sugar/yeast food and then for newly emerged flies to feed on the same nutritionally complete diet for 48 hr to standardize mating conditions before they are allocated to experimental diets for the remainder of their lives. It is thus possible that adult flies can acquire and store all they need of some nutrients during the 48‐hr window after emerging to support the rest of adult life. To investigate this, we first assessed survival of flies on cholesterol dropout diet when they had access to a nutritionally complete holidic diet for zero, 2 or 4 days immediately after eclosing as adults (Figure 3b and Figure S4d). We found that survival of mated females and *ovo^D1^* females was increased with each increment of time of exposure to a complete diet, and that for males, only depriving cholesterol from eclosion (day 0) very slightly decreased lifespan (Figure 3b and Figure S4d). A similar result was observed when flies were deprived of vitamins from the time of eclosion (Figure 3b and Figure S4e). However, removal of choline, myo‐inositol, inosine, uridine, and metal ions from the diet of flies as they eclosed did not compromise survival of males or *ovo^D1^* sterile females (Figure 3b and Figure S4f). Thus, adult males exhibit a minor survival benefit when permitted to supplement their larval acquired stores of cholesterol and vitamins immediately after eclosion, but all requirements for choline, inositol, uridine, inosine, and metals are carried over from development—a fact that is also true for sterile females. This is consistent with the finding that most adult nutrition is committed directly to reproduction (Min, Hogan, Tatar, & O'Brien, 2006).

## 3 DISCUSSION

Together, our data capture the minimal nutritional requirements of adult flies for full lifespan, which in past studies we have shown to equal the maximum achievable for flies on a laboratory‐based diet that was optimized for lifespan using natural ingredients (Bass et al., 2007; Piper et al., 2014). Remarkably, males only require an ongoing source of sugar and amino acids, but females have a strong additional dependency upon dietary cholesterol and vitamins throughout life. These needs were found to be greater in reproducing females than for those that were not reproducing. In combination with the range of concentrations of each of these nutrients likely to be found in fly diets (Lange & Heijnen, 2001; Piper, 2017), these data provide boundaries for studies that seek to define how changes in adult nutrition affect healthy lifespan outcomes using *Drosophila*.

DR or altered DB can produce large changes in lifespan of mated female flies and less so in males and nonreproducing females (Chapman & Partridge, 1996; Magwere et al., 2004; Mair et al., 2004). This is consistent with an argument that the higher costs of reproduction in fertile females over that in males and nonreproducing females deprive somatic maintenance of resources, and this results in shortened lifespan (Kirkwood, 2002). But our previous work has shown that rebalancing the proportion of dietary amino acids can provide a single nutritional optimum for maximal lifespan and maximal reproduction (Piper et al., 2017), indicating that rather than lifespan being governed by an obligate trade‐off with reproduction, there is some other reason for why chronic exposure to high levels of food shortens lifespans. More recent work to alter DB, instead of simply restricting all nutrients as in DR, indicates that this is caused by chronic ingestion of high protein, low carbohydrate diets (Lee et al., 2008; Mair et al., 2005; Skorupa et al., 2008; Solonbiet et al., 2014)—an effect that has been summarized in the lethal protein hypothesis (Fanson et al., 2012; Raubenheimer & Simpson, 2009; Sanz, Caro, & Barja, 2004; Simpson & David, 2009; Victoria et al., 2007).

Almost all nutrient explicit studies on *Drosophila* lifespan have focused on the effects of varying the relative proportions of sugar and protein in the diet. While our data indicate that this is justified for males, we also show that other nutrients are important for determining female lifespan. Considering these other factors is important because in work using diet change to study aging, we assume that all diet manipulations that prolong fly lifespan are due to the same underlying mechanisms and so comparable. However, our data here indicate that while it is possible that high dietary protein may shorten lifespan by direct toxicity, it may also shorten lifespan through some indirect effect of raising fecundity and thus creating a deficit in cholesterol or vitamins. The approach that we have taken of manipulating a single dietary nutrient, while holding all others constant, is inadequate to distinguish between these possibilities. In order to detect these, more complex nutrient interactions and their differing mechanistic bases require additional experiments that use systematic changes in combinations of dietary components according to the methodology of Nutritional Geometry (Simpson & Raubenheimer, 1993, 2012). Understanding these interactions is critical for how we interpret the published mechanistic studies of how diet modifies lifespan.

## 4 EXPERIMENTAL PROCEDURES

### 4.1 Fly stocks and husbandry

The wild‐type stock *Dahomey* was collected in 1970 in Dahomey (now Benin). Canton S stock was gift from Haihuai He (Sichuan University). *w1118*, 4,248 (C(1)RM, y[1] pn[1] v[1]/C(1;Y)1, y[1] B[1]/0; sv[spa‐pol]), and 1,309(ovo[D1] v[24]/C(1)DX, y[1] w[1] f[1]) were obtained from the Bloomington Stock Center. The males of 4,248 were crossed with *Dahomey* female to obtain XO males (sterile) and XXY females. The males of 1,309 were crossed with either *Dahomey* or Canton S females to obtain sterile *ovo^D1^* females. All stocks were maintained at 25°C on a 12‐hr: 12‐hr light:dark cycle at constant humidity using 1SY food (10 g agar/50 g sucrose/100 g yeast [Yeast brand: ANGEL YA100]), but experiments were conducted on holidic medium (with amino acids in the ratio of Yaa (Piper et al., 2014)) at 25°C on a 12‐hr:12‐hr light:dark cycle at constant humidity. For all experiments, flies were reared at standard larval density in 1SY food and eclosed adults were collected over a 12‐hr period (Piper & Partridge, 2016). Flies were mated for 48 hr on 1SY food in all experiments (except for Figure 3b, which flies were mated for 48 hr on holidic medium) before sorting into single sexes. 100N50S Yaa medium (Piper et al., 2014) was used as control diet in all experiments.

### 4.2 Lifespan analysis

Flies were randomly allocated to the experimental food treatments and housed in plastic vials containing food at a density of 10 flies per vial, with 10 vials per condition (*n* = 100). Flies were transferred to a fresh food source every 2–3 days, during which any deaths and censors were recorded. Lifespan differences were assessed using the Cox regression and log‐rank test (see Supplemental Tables).

### 4.3 Fecundity

The number of eggs laid in 24‐hr periods (days 7–8) was counted in all experiment except Figure 2e,f. In Figure 2e,f, egg numbers in 24‐hr periods were counted every 2–3 days from day 2–day 24, and data are reported as cumulative eggs laid per female in figure. For each condition and each time point, 10 vials were counted. Each vial contained 10 flies. Egg‐laying differences were assessed by one‐way ANOVA followed by Tukey's multiple comparison or by two‐way ANOVA followed by Sidak's multiple comparisons test.

## CONFLICT OF INTEREST

No competing interest declared.

## AUTHOR CONTRIBUTIONS

QW and MY conceived the experiments; QW, GY, XC, YG, XF, and DY performed experiments. MX and TW contributed reagents/materials. All authors analyzed the data. QW, MY, and MP wrote the manuscript. All authors provided final approval of the submitted version.

## Data Availability

The data that support the findings of this study are openly available in Figshare at https://doi.org/10.26180/5db6e11d52f79, reference number: doi.org/10.26180/5db6e11d52f79 (Wu et al., 2019).
