# Supplementary material for: Sexual dimorphism in the nutritional requirement for adult lifespan in Drosophila melanogaster
Source: Aging Cell. 2020 Feb 18;19(3):e13120. doi: 10.1111/acel.13120 (PMC7059147; doi:10.1111/acel.13120)
Supplement: Supplementary file 1 [file ACEL-19-e13120-s001.pdf]

Supplemental Information

Sexual dimorphism in the nutritional requirement for adult lifespan in *Drosophila melanogaster*

Qi Wu, Guixiang Yu, Xingyi Cheng, Yue Gao, Xiaolan Fan, Deying Yang, Meng Xie, Tao Wang, Matthew D.W. Piper, Mingyao Yang

Supplemental Data

Figure S1. Lifespan curves of Figure 1c-g
Figure S2. Lifespan curves of Figure 2a-f.
Figure S3 Lifespan curves.
Figure S4 Lifespan curves of Figure 3a-b.
Figure S5 Lifespan curves.
Table S1. Lifespan data for Figure S1a.
Table S2. Lifespan data for Figure S1b.
Table S3. Lifespan data for Figure S1c.
Table S4. Lifespan data for Figure S1d.
Table S5. Lifespan data for Figure S1e.
Table S6. Lifespan data for Figure S2a.
Table S7. Lifespan data for Figure S2b.
Table S8. Lifespan data for Figure S2c
Table S9. Lifespan data for Figure S2d.
Table S10. Lifespan data for Figure S2e.
Table S11. Lifespan data for Figure S2f.
Table S12. Lifespan data for Figure S3a
Table S13. Lifespan data for Figure S3b.
Table S14. Lifespan data for Figure S4b.
Table S15. Lifespan data for Figure S4c.
Table S16. Lifespan data for Figure S4d.
Table S17. Lifespan data for Figure S4e.
Table S18. Lifespan data for Figure S4f.
Table S19. Lifespan data for Figure S5

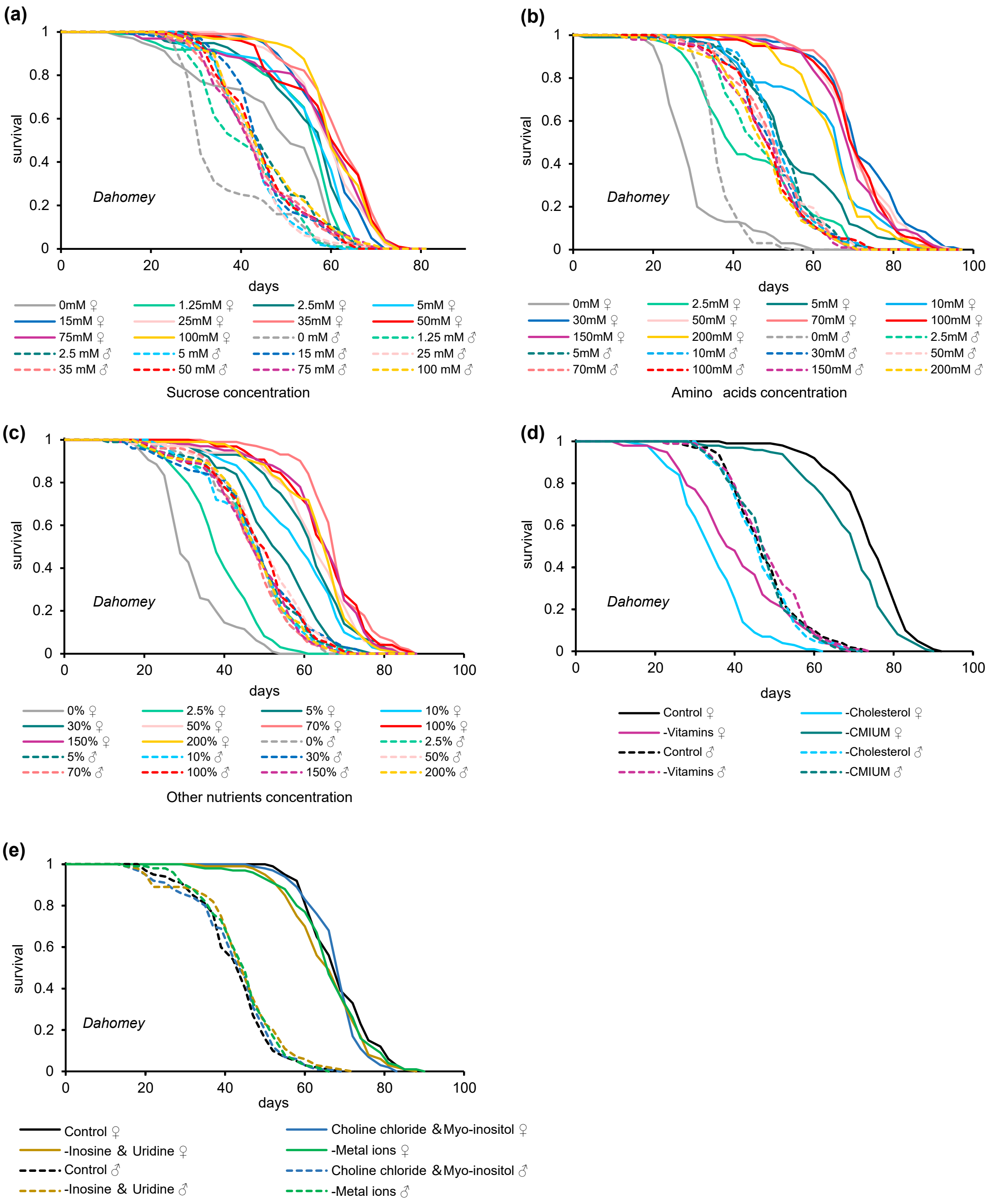

Figure S1. Lifespan curves of Figure 1c-g. (a-c) Effect of manipulating sugar **(a)**, total amino acid **(b)** and other nutrients level **(c)** on lifespan in both sex. **(d)** The effect of removing dietary cholesterol, B-group vitamins or other ingredients include choline, myo-inositol, uridine, inosine and metal ions (labeled as CMIUM in the figure) on lifespan in both sex. **(e)** Effect of omitting choline and myo-inositol (C&M), inosine and uridine (I&U) or metal ions on female day 8 fecundity and lifespan in both sex of dahomey strain. (n = 100 flies per treatment for lifespans, see statistical analysis of lifespan data in Supplemental Table S1-S8)

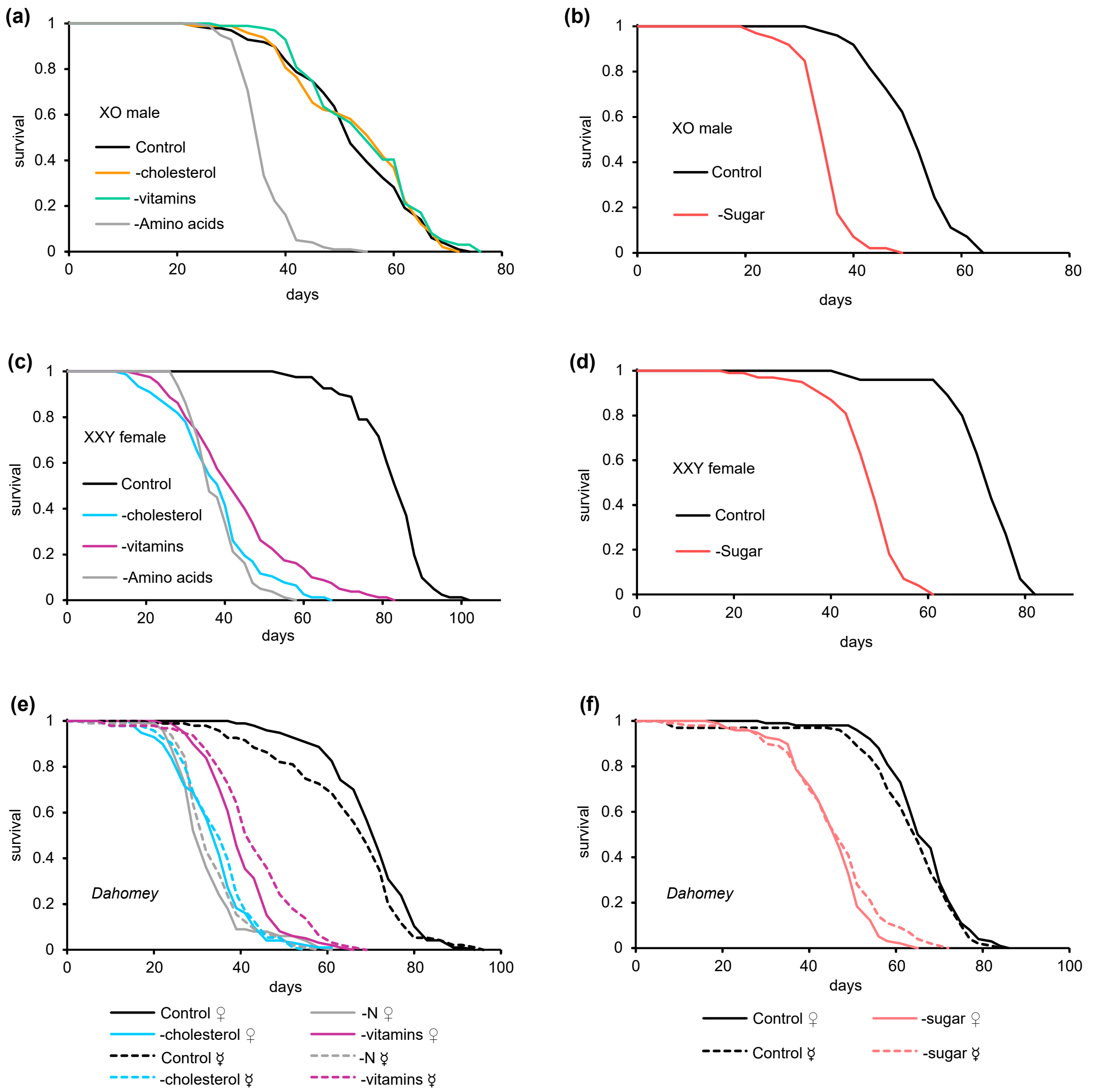

Figure S2. Lifespan curves of Figure 2a-f.

(a-b) Effect of omitting amino acid (N), cholesterol (chol), B-group vitamins (vit) or sucrose (S) on lifespan of XO males. **(c-d)** Effect of omitting N, chol, vit or S on lifespan of XXY females. **(e-f)** Effect of omitting N, chol, vit or S on lifespan of mated wild type *Dahomey* females (♀) and virgin females (☿). (n = 100 flies per treatment for lifespans, see statistical analysis of lifespan data in Supplemental Table S6-S11)

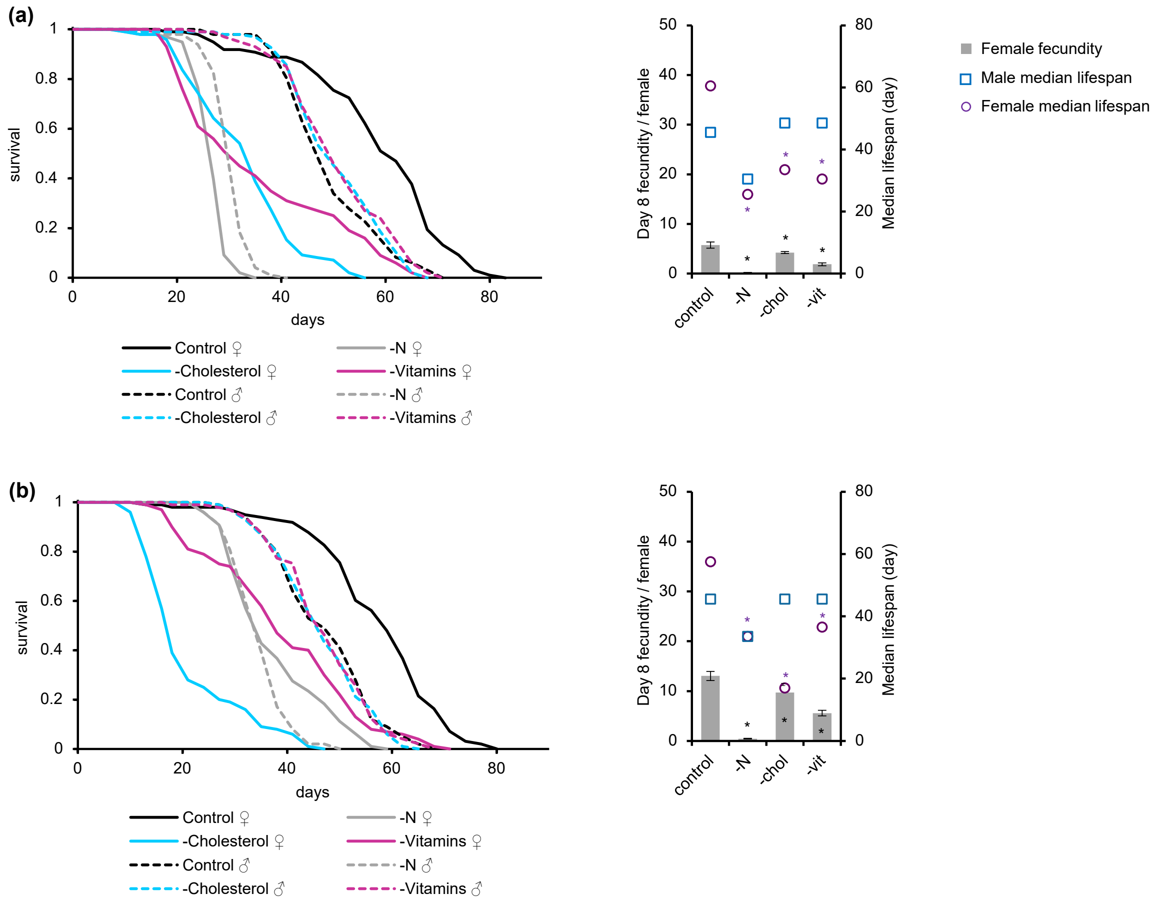

Figure S3. Lifespan curves. (a-b) Effect of omitting amino acids, cholesterol or B-group vitamins on female day 8 fecundity and lifespan in both sex of *w1118* **(a)** and *Canton S* **(b)** strains. (n = 100 flies per treatment for lifespans and n = 10 biological replicates for egg laying in all trials, error bars represent egg-laying mean ± s.e.m. Within each phenotype,* indicate $P < 0.05$ versus complete holidic diet control. Lifespan differences were assessed using Cox regression and egg-laying differences were assessed by one-way ANOVA followed by Tukey's multiple comparison. See statistical analysis of lifespan data in Supplemental Table S12-S13)

.

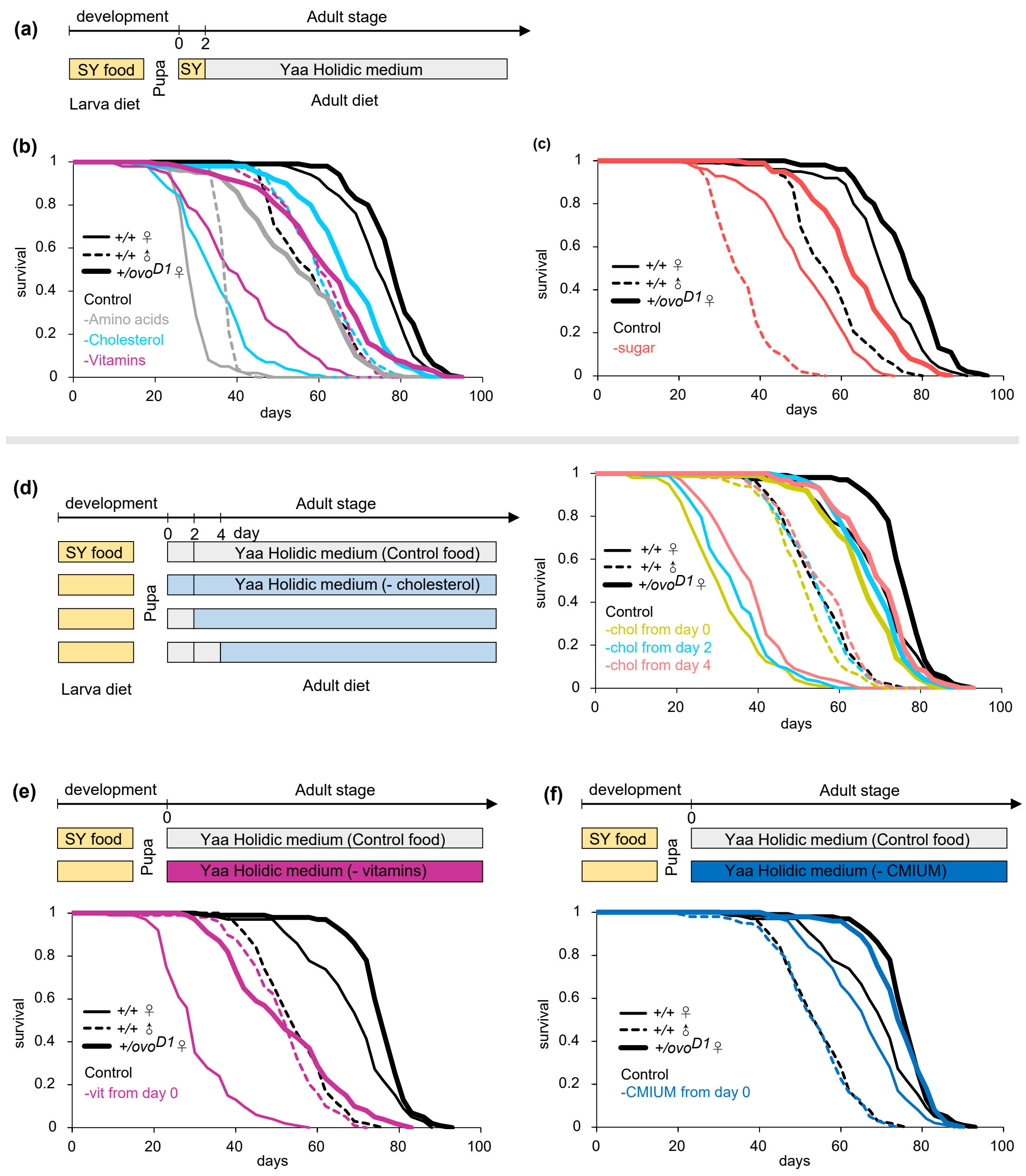

Figure S4. Lifespan curves of Figure 3a-b.

(a-c) Effect of omitting amino acids (N), cholesterol (chol), B-group vitamins (vit) or sucrose (S) from the adult diet from day 2 post-eclosion on lifespan of wild type females and males and ovo^{D1} mutant females. **(d)** Effect of omitting cholesterol in adult diet from day 0, 2, 4 post-eclosion on lifespan of wild type females and males and ovo^{D1} mutant females. (**e-f**) Effect of omitting vitamins **(e)** or other components **(f)** include choline, myo-inositol, uridine, inosine, metals (labeled as CMIUM in the figure) in adult diet immediately from post-eclosion on lifespan of wild type females and males and ovo^{D1} mutant females. ovo[D1] v[24]/C(1)DX, y[1] w[1] f[1] males (Bloomington stock number 1309) were crossed with Dahomey females to obtain +/ovo^{D1} ♀ and +/+ ♂; +/+ ♀ in the Figure were Dahomey females. n = 100 flies per treatment for lifespans, see statistical analysis of lifespan data in Supplemental Table S14-S18)

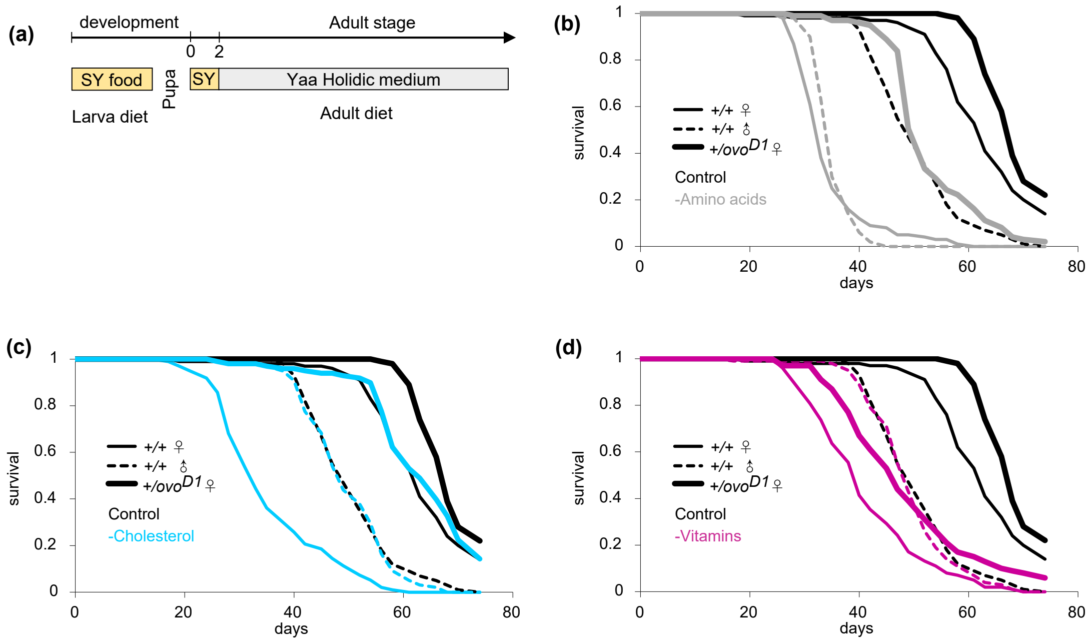

Figure S5. Lifespan curves.

ovo^{D1} mutant lifespan curves of another genetic background.

(a-d) Effect of omitting amino acids **(b)**, cholesterol **(c)**, B-group vitamins **(d)** from the adult diet from day 2 post-eclosion on lifespan of wild type females and males and *ovo*^{D1} mutant females. (ovo[D1] v[24]/C(1)DX, y[1] w[1] f[1]) (Bloomington stock 1309) males were crossed with Canton S females to obtain +/*ovo*^{D1} ♀ and +/+ ♂; +/+ ♀ in the Figure were Canton S females. n = 100 flies per treatment for lifespans, see statistical analysis of lifespan data in Supplemental Table S19)

Table S1. Lifespan data for Figure S1a.

Females										
Sucrose concentration	0mM	1.25mM	2.5mM	5mM	15mM	25mM	35mM	50mM (control)	75mM	100mM
Number of death	101	98	97	99	100	97	101	97	99	96
Number of censor	0	1	0	0	0	2	0	0	1	1
median (d)	49.5	55.5	58.5	58.5	61.5	61.5	62.5	61.5	61.5	61.5
mean (d)	47.8	53.4	54.9	55.4	59.5	60.1	62.3	60.0	58.3	61.6
maximum (d)	58.5	61.5	64.5	64.5	67.5	70.5	70.5	70.5	70.5	70.5
log-rank (vs control)	1.64E-12	2.21E-09	7.61E-07	2.90E-06	0.04853	0.22323	0.46135		0.35854	0.88431
Cox reg (vs control)	6.66E-15	1.67E-09	2E-06	0.00001	0.06657	0.24275	0.54105		0.45343	0.85094
Males										
Sucrose concentration	0mM	1.25mM	2.5mM	5mM	15mM	25mM	35mM	50mM (control)	75mM	100mM
Number of death	100	98	100	97	99	98	97	97	93	100
Number of censor	0	2	0	3	1	1	0	1	1	0
median (d)	26.5	35.5	41.5	40	41.5	40	40	40	40	40
mean (d)	33.6	39.0	43.3	40.7	42.9	40.8	41.4	42.4	41.5	43.3
maximum (d)	52.5	52.5	61.5	52.5	58.5	52.5	58.5	58.5	60	61.5
log-rank (vs control)	1.28E-06	0.04022	0.37303	0.04779	0.82308	0.1656	0.67211		0.83317	0.39777
Cox reg (vs control)	9.85E-07	0.07050	0.48508	0.14052	0.88259	0.19101	0.77221		0.86408	0.44713
Females and Males										
Cox regression (sucrose)			p < 0.0001							
Cox regression (gender)			p < 0.0001							
Cox regression (sucrose×gender)			p = 0.0003							

Table S2. Lifespan data for Figure S1b.

Females										
Amino acids concentration	0mM	2.5mM	5mM	10mM	30mM	50mM	70mM	100mM (control)	150mM	200mM
Number of death	100	99	100	100	98	100	99	100	98	98
Number of censor	0	0	0	0	2	0	1	0	2	2
median (d)	28	39.5	51	66	70	70	70	70	68	66
mean (d)	30.2	45.2	55.5	62.9	72.8	71.4	71.5	70.4	69.8	65.3
maximum (d)	50	68	77.5	82	89	84.5	81	82	87	83
log-rank (vs control)	1.54E-49	5.29E-28	1.09E-10	0.00121	0.05532	0.53397	0.98101		0.5588	0.0002
Cox reg (vs control)	3.44E-94	9.06E-31	3.30E-10	0.00461	0.10408	0.58151	0.93844		0.76359	0.00260
Males										
Amino acids concentration	0mM	2.5mM	5mM	10mM	30mM	50mM	70mM	100mM (control)	150mM	200mM
Number of death	98	96	100	98	98	98	99	98	97	96
Number of censor	1	2	0	2	2	2	0	1	3	4
median (d)	34.5	46	51	51	48.5	51	51	48.5	48.5	46
mean (d)	36.7	47.6	52.6	52.0	50.0	50.4	50.2	49.3	48.2	47.5
maximum (d)	44	65	65	63	69	63	64	65	63	64
log-rank (vs control)	1.56E-25	0.26459	0.06277	0.16848	0.90348	0.50271	0.68805		0.59805	0.27663
Cox reg (vs control)	8.14E-32	0.27016	0.13287	0.31148	0.91619	0.59452	0.83564		0.47737	0.28751
Females and Males										
Cox regression (amino acids)		p < 0.0001								
Cox regression (gender)		p < 0.0001								
Cox regression (amino acids×gender)		p < 0.0001								

Table S3. Lifespan data for Figure S1c.

Females										
Other nutrients concentration	0%	2.50%	5%	10%	30%	50%	70%	100% (control)	150%	200%
Number of death	96	97	99	100	100	100	101	97	100	103
Number of censor	1	0	0	0	0	0	0	2	1	0
median (d)	27	37	53	57.5	62	62	67	64.5	64.5	64.5
mean (d)	32.6	39.3	53.3	58.3	61.1	62.5	68.2	65.1	64.6	63.6
maximum (d)	49	51	66	76	78	76	83	78	77	76
log-rank (vs control)	1.90E-46	3.53E-41	7.74E-14	0.00021	0.01154	0.09396	0.13455		0.64654	0.12646
Cox reg (vs control)	1.86E-86	7.09E-61	1.33E-14	0.00033	0.01796	0.14846	0.21677		0.71038	0.19596
Males										
Other nutrients concentration	0%	2.50%	5%	10%	30%	50%	70%	100% (control)	150%	200%
Number of death	100	99	99	100	99	100	100	100	98	99
Number of censor	0	0	0	0	0	0	0	0	1	1
median (d)	46.5	46.5	49	46.5	49	49	46.5	49	46.5	46.5
mean (d)	47.3	47.8	48.6	47.4	48.5	50.3	47.4	49.0	47.5	48.0
maximum (d)	60	64.5	64.5	62	68	67	60	64.5	59	61
log-rank (vs control)	0.06458	0.41039	0.59274	0.22325	0.83381	0.58715	0.04877		0.17203	0.23679
Cox reg (vs control)	0.11111	0.43670	0.63227	0.26179	0.77260	0.66141	0.08306		0.21959	0.31061
Females and Males										
Cox regression (other nutrients)	$p < 0.0001$									
Cox regression (gender)	$p < 0.0001$									
Cox regression (other nutrients × gender)	$p < 0.0001$									

Table S4. Lifespan data for Figure S1d.

	Female				Male			
food type	control	-cholesterol	-vitamins	-CMIUM	control	-cholesterol	-vitamins	-CMIUM
Number of death	100	100	96	98	100	100	100	99
Number of censor	0	0	2	1	0	0	0	0
median (d)	75	34.5	38	70.5	46	46	47	46
mean (d)	74.6	35.1	41.2	69.2	48.2	47.2	48.9	47.9
maximum (d)	86	49	61	82	65	59	62	61
log-rank (vs control)		1.85E-51	1.99E-46	0.00092		0.38543	0.58864	0.64000
Cox reg (vs control)		6.46E-59	2.00E-46	0.00370		0.36754	0.63185	0.66676
Cox regression (cholesterol × gender)			p < 0.0001					
Cox regression (vitamins × gender)			p < 0.0001					
Cox regression (CMIUM × gender)			p = 0.485					

Table S5. Lifespan data for Figure S1e.

	Female				Male			
food type	Control	-Choline &inositol	-Inosine &Uridine	-Metal ions	Control	-Choline &inositol	-Inosine &Uridine	-Metal ions
Number of death	100	100	100	100	100	100	100	100
Number of censor	0	0	0	0	0	0	0	0
median (d)	67.5	67.5	64.5	64.5	43.5	43.5	43.5	43.5
mean (d)	68.9	68.4	66.2	66.8	43.1	43.0	44.6	44.7
maximum (d)	81	77.5	75	80	58	58	61.5	53.5
log-rank (vs control)		0.19544	0.09399	0.35887		0.64696	0.11436	0.28666
Cox reg (vs control)		0.31312	0.14043	0.38626		0.67957	0.14475	0.32686

Table S6. Lifespan data for Figure S2a.

	XO male			
food type	Control	-cholesterol	-vitamins	-Amino acids
Number of death	99	99	99	100
Number of censor	1	0	1	0
median (d)	50.5	56.5	53.5	34.5
mean (d)	53.0	53.7	54.9	36.6
maximum (d)	66	68	66	41
log-rank (vs control)		0.68349	0.20064	1.32E-31
Cox reg (vs control)		0.67751	0.23459	2.51E-34

Table S7. Lifespan data for Figure S2b.

	XO male	
food type	Control	-sucrose
Number of death	98	98
Number of censor	1	0
median (d)	50.5	35.5
mean (d)	51.7	35.5
maximum (d)	62.5	38.5
log-rank (vs control)		3.85E-40
Cox reg (vs control)		3.92E-27

Table S8. Lifespan data for Figure S2c.

	XXY female			
food type	Control	-cholesterol	-vitamins	-Amino acids
Number of death	81	77	80	80
Number of censor	0	3	0	0
median (d)	84.5	39	41	34.5
mean (d)	82.7	38.4	43.9	38.5
maximum (d)	90	59	69	46
log-rank (vs control)		6.21E-43	2.51E-38	1.27E-43
Cox reg (vs control)		2.98E-36	2.07E-31	6.85E-38

Table S9. Lifespan data for Figure S2d.

	XXY female	
food type	Control	-sucrose
Number of death	100	100
Number of censor	0	0
median (d)	71.5	47.5
mean (d)	72.5	48.3
maximum (d)	77.5	53.5
log-rank (vs control)		1.18E-45
Cox reg (vs control)		4.22E-16

Table S10. Lifespan data for Figure S2e.

	Mated female (♀)				Virgin female (☿)			
food type	control	-Amino acids	-cholesterol	-vitamins	control	-Amino acids	-cholesterol	-vitamins
Number of death	97	100	99	99	95	97	94	95
Number of censor	3	0	0	1	5	2	4	5
median (d)	70.5	30.5	33.5	38	67.5	30.5	33.5	42
mean (d)	70.6	32.8	34.1	40.4	65.3	33.9	35.0	43.7
maximum (d)	81.5	41	44.5	50.5	78.5	47.5	44.5	56.5
Cox reg (vs control)		1.15E-42	4.94E-38	3.44E-28		9.27E-40	3.33E-35	1.23E-20
log-rank (vs control)		8.04E-49	1.98E-47	1.13E-44		6.29E-40	5.77E-38	4.50E-27
log-rank (♀vs ☿)					0.06500	0.37822	0.45305	0.00388
Cox regression (amino acids × mating status)				p = 0.107				
Cox regression (cholesterol × mating status)				p = 0.122				
Cox regression (vitamins × mating status)				p = 0.0025				

Table S11. Lifespan data for Figure S2f.

	Mated female (♀)		Virgin female (☿)	
food type	Control	-sugar	Control	-sugar
Number of death	100	98	100	100
Number of censor	0	2	0	0
median (d)	65	45.5	64	45.5
mean (d)	66.4	45.4	63.5	46.5
maximum (d)	78	55	76	64
Cox reg (vs control)		3.15E-31		2.82E-20
log-rank (vs control)		4.23E-41		1.36E-24
log-rank (♀vs ☿)			0.18463	0.07534
Cox regression (sucrose × mating status)			p = 0.272	

Table S12. Lifespan data for Figure S3a.

	Female				Male			
food type	control	-Amino acids	-cholesterol	-vitamins	control	-Amino acids	-cholesterol	-vitamins
Number of death	98	98	98	100	97	98	97	100
Number of censor	0	0	0	0	2	1	2	1
median (d)	60.5	25.5	33.5	30.5	45.5	30.5	48.5	48.5
mean (d)	59.1	27.1	33.4	36.1	49.8	30.8	50.9	51.2
maximum (d)	75.5	28	48.5	60.5	63.5	33.5	63.5	63.5
Cox reg (vs control)		5.07E-37	1.37E-24	6.62E-19		1.82E-43	0.65694	0.28456
log-rank (vs control)		1.57E-40	7.10E-34	3.81E-20		2.59E-44	0.59601	0.23735

Table S13. Lifespan data for Figure S3b.

	Female				Male			
food type	control	-Amino acids	-cholesterol	-vitamins	control	-Amino acids	-cholesterol	-vitamins
Number of death	98	98	100	100	100	99	99	97
Number of censor	1	0	0	0	1	1	0	2
median (d)	57.5	33.5	17	36.5	45.5	33.5	45.5	45.5
mean (d)	57.8	37.7	21.3	39.4	47.6	35.0	47.1	47.4
maximum (d)	69.5	54.5	38	60.5	60.5	39.5	57.5	59
Cox reg (vs control)		1.06E-20	2.99E-55	3.67E-14		7.34E-26	0.51884	0.81505
log-rank (vs control)		4.94E-29	1.59E-46	5.36E-17		9.94E-25	0.43908	0.82455

Table S14. Lifespan data for Figure S4b.

	+/+ female				+/+ male				+/ovo^{D1} female			
food type	control	-Amino acids	-cholesterol	-vitamins	control	-Amino acids	-cholesterol	-vitamins	control	-Amino acids	-cholesterol	-vitamins
Number of death	100	99	100	96	100	100	100	100	100	100	100	100
Number of censor	0	1	0	4	0	0	0	0	0	0	0	0
median (d)	75	27	34.5	38	56.5	37	59	60	77.5	53.5	66	62
mean (d)	74.6	30.0	35.1	41.2	58.5	37.9	61.2	60.6	78.4	55.1	66.6	61.8
maximum (d)	86	31.5	49	61	70.5	39	75	73	89	73	79	82
Cox reg (vs control)		4.57E-52	4.32E-39	7.39E-31		2.28E-38	0.16735	0.34955		2.52E-29	1.36E-9	5.62E-13
log-rank (vs control)		1.81E-51	1.85E-51	1.99E-46		2.85E-47	0.12102	0.29790		1.62E-36	2.14E-15	8.84E-16
log-rank (vs +/+ female)					7.32E-26	4.83E-27	2.16E-43	2.85E-22	0.00229	2.76E-40	1.36E-45	3.77E-22
log-rank (vs +/+ male)									1.28E-35	9.41E-34	4.60E-06	0.02110
+/+ female vs +/ovo^{D1} females: Cox regression (amino acids × genotype)	$p < 0.0001$											
+/+ female vs +/ovo^{D1} females: Cox regression (cholesterol × genotype)	$p < 0.0001$											
+/+ female vs +/ovo^{D1} females: Cox regression (vitamins × genotype)	$p < 0.0001$											
+/+ female vs +/+ males: Cox regression (amino acids × gender)	$p < 0.0001$											
+/+ female vs +/+ males: Cox regression (cholesterol × gender)	$p < 0.0001$											
+/+ female vs +/+ males: Cox regression (vitamins × gender)	$p < 0.0001$											

Table S15. Lifespan data for Figure S4c.

	+/+ female		+/+ male		+/*ovo*^{D1} female	
food type	Control	-sucrose	Control	-sucrose	Control	-sucrose
Number of death	100	98	97	100	100	100
Number of censor	0	2	3	0	0	0
median (d)	70	50	54.5	34	76	62
mean (d)	70.6	51.2	57.6	36.2	76.6	64.2
maximum (d)	83	67	71.5	47	88	80
Cox reg (vs control)		5.13E-25		5.44E-32		8.67E-13
log-rank (vs control)		2.09E-31		4.39E-42		1.81E-15
log-rank (vs +/+ female)			2.29E-19	6.36E-25	6.91E-06	9.30E-15
log-rank (vs +/+ male)					1.84E-32	2.99E-48
+/+ female vs +/*ovo*^{D1} females: Cox regression (sucrose × genotype)				p = 0.0002		
+/+ female vs +/+ males: Cox regression (sucrose × gender)				p = 0.0049		

Table S16. Lifespan data for Figure S4d.

		+/+ female				+/+ male				+/*ovo*^{D1} female			
food type		Control	-chol from day 0	-chol from day 2	-chol from day 4	Control	-chol from day 0	-chol from day 2	-chol from day 4	Control	-chol from day 0	-chol from day 2	-chol from day 4
Number of death		98	97	98	100	100	99	98	100	100	100	100	100
Number of censor		1	2	0	0	0	1	1	0	0	0	0	0
median (d)		70.5	29	34.5	38	53.5	50.5	53.5	55	75	66	68	70.5
mean (d)		69.0	32.1	34.8	38.4	55.1	51.7	54.5	55.7	76.0	65.8	68.5	69.9
maximum (d)		82	48	50.5	53.5	68	66	66	66	84.5	77.5	80	79
+/+ female	Control		1.15E-48	2.06E-46	1.20E-43	2.50E-21	2.61E-28	1.13E-22	2.00E-20	0.00005	4.12E-03	0.16108	0.94652
	-chol from day 0			0.08420	8.71E-05	1.21E-36	6.82E-30	3.74E-35	1.29E-36	2.80E-52	4.09E-48	1.06E-51	3.63E-51
	-chol from day 2				0.01529	1.70E-32	4.15E-25	1.17E-30	9.48E-33	7.99E-52	2.68E-46	2.36E-50	5.60E-50
	-chol from day 4					1.04E-23	4.90E-17	7.90E-22	6.64E-25	2.36E-51	5.98E-42	1.09E-46	5.38E-47
+/+ male	Control						0.00699	0.55879	0.49646	1.50E-42	2.19E-15	7.14E-21	2.69E-24
	-chol from day 0							0.02123	0.00052	5.42E-47	2.17E-23	3.12E-30	2.13E-32
	-chol from day 2								0.18524	3.07E-44	3.54E-17	3.16E-23	1.17E-26
	-chol from day 4									6.60E-43	3.96E-14	1.55E-19	1.90E-23
+/OVO^{D1} female	Control										3.82E-14	8.96E-10	9.19E-06
	-chol from day 0											0.10800	0.00222
	-chol from day 2												0.12089
	-chol from day 4												

Table S17. Lifespan data for Figure S4e.

	+/+ female		+/+ male		+/*ovo*^{D1} female	
food type	Control	-vitamins	Control	-vitamins	Control	-vitamins
Number of death	99	100	100	99	100	100
Number of censor	1	0	0	1	0	0
median (d)	70.5	29	53.5	50.5	75	50.5
mean (d)	69.0	30.9	55.1	52.1	76.0	52.4
maximum (d)	82	47	68	67	84.5	74
Cox reg (vs control)		7.36E-32		0.07340		1.76E-24
log-rank (vs control)		2.38E-49		0.04379		2.32E-30
log-rank (vs +/+ female)			2.50E-21	1.15E-33	5.13E-05	5.22E-29
log-rank (vs +/+ male)					1.50E-42	0.09126

Table S18. Lifespan data for Figure S4f.

	+/+ female		+/+ male		+/*ovo*^{D1} female	
food type	Control	-CMIUM	Control	-CMIUM	Control	-CMIUM
Number of death	99	100	100	98	100	100
Number of censor	1	0	0	0	0	0
median (d)	70.5	66	53.5	53.5	75	74
mean (d)	69.0	65.8	55.1	54.0	76.0	74.7
maximum (d)	82	80	68	66	84.5	82
Cox reg (vs control)		0.03557		0.64039		0.50707
log-rank (vs control)		0.01903		0.60151		0.43956
log-rank (vs +/+ female)			2.50E-21	1.18E-15	5.13E-05	9.08E-09
log-rank (vs +/+ male)					1.50E-42	3.00E-39

Table S19. Lifespan data for Figure S5b-d.

	+/+ female				+/+ male				+/OVO^{D1} female			
food type	control	-Amino acids	-cholesterol	-vitamins	control	-Amino acids	-cholesterol	-vitamins	control	-Amino acids	-cholesterol	-vitamins
Number of death	86	100	97	99	100	100	98	100	78	97	84	94
Number of censor	0	0	1	0	0	0	2	0	0	1	2	0
median (d)	62	32	32	39	48	34	48	48	67	50.5	62	46
mean (d)	51.9	34.8	35.5	41.3	50.7	35.3	49.9	49.5	51.7	51.4	52.3	44.1
maximum (d)		46	53	59.5	64.5	39	59.5	62		67		
log-rank (vs control)		1.81E-44	1.13E-42	8.70E-31		4.13E-45	0.49032	0.31239		2.80E-26	0.00421	5.74E-21
log-rank (vs +/+ female)						0.27358	4.18E-17	4.01E-06		1.50E-33	4.38E-42	1.32E-05
log-rank (vs +/+ male)										1.05E-46	4.06E-24	0.59285
